# {4,6-Bis[(*E*)-1-methyl-2-(pyridin-2-yl­methyl­idene-κ*N*)hydrazinyl-κ*N* ^2^]pyrimidine-κ*N* ^1^}dichloridocopper(II) methanol disolvate monohydrate

**DOI:** 10.1107/S1600536811025414

**Published:** 2011-07-09

**Authors:** Bartosz Marzec, M. Baby Mariyatra, Thomas McCabe, Wolfgang Schmitt

**Affiliations:** a School of Chemistry & CRANN, The University of Dublin, Trinity College, Dublin 2, Ireland

## Abstract

The title compound, [CuCl_2_(C_18_H_18_N_8_)]·2CH_3_OH·H_2_O, contains a penta­coordinated Cu(II) atom bonded to the tridentate 4,6-bis­[(*E*)-1-methyl-2-(pyridin-2-yl­methyl­idene)hydrazin­yl]pyrimidine ligand and two Cl atoms. The geometry around the Cu^II^ atom is distorted square-pyramidal. The mol­ecules pack in the crystal structure *via* O—H⋯Cl, O—H⋯N, C—H⋯Cl and C—H⋯O hydrogen bonds, C—H⋯π and π–π inter­actions [centroid–centroid distances of the pyrimidine–pyridine and pyridine–pyridine inter­actions are 3.750 (3) and 3.850 (3) Å, respectively], forming sheet-like assemblies.

## Related literature

For the coordination chemistry of similar ligand-types, see: Stadler *et al.* (2005 ▶, 2006 ▶). For additional geometric analysis, see: Addison *et al.* (1984 ▶).
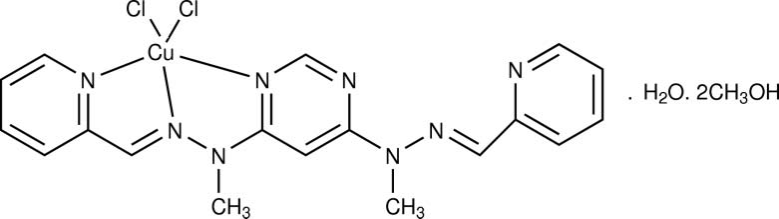


## Experimental

### 

#### Crystal data

[CuCl_2_(C_18_H_18_N_8_)]·2CH_4_O·H_2_O
*M* *_r_* = 560.94
Triclinic, 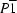

*a* = 7.430 (5) Å
*b* = 11.627 (8) Å
*c* = 14.026 (9) Å
α = 95.848 (7)°
β = 93.477 (13)°
γ = 92.920 (9)°
*V* = 1201.2 (14) Å^3^
*Z* = 2
Mo *K*α radiation
μ = 1.17 mm^−1^
*T* = 116 K
0.30 × 0.25 × 0.10 mm

#### Data collection

Rigaku Saturn724 diffractometer
Absorption correction: multi-scan (*CrystalClear*; Rigaku, 2008 ▶) *T*_min_ = 0.786, *T*_max_ = 1.000
26094 measured reflections
7050 independent reflections
4093 reflections with *I* > 2σ(*I*)
*R*_int_ = 0.091

#### Refinement

*R*[*F* ^2^ > 2σ(*F* ^2^)] = 0.047
*wR*(*F* ^2^) = 0.104
*S* = 0.82
7050 reflections
311 parameters
H-atom parameters constrained
Δρ_max_ = 1.20 e Å^−3^
Δρ_min_ = −0.64 e Å^−3^

### 

Data collection: *CrystalClear* (Rigaku, 2008 ▶); cell refinement: *CrystalClear*; data reduction: *CrystalClear*; program(s) used to solve structure: *SHELXS97* (Sheldrick, 2008 ▶); program(s) used to refine structure: *SHELXL97* (Sheldrick, 2008 ▶); molecular graphics: *ORTEP-3 for Windows* (Farrugia, 1997 ▶), *DIAMOND* (Brandenburg, 1998 ▶) and *PLATON* (Spek, 2009 ▶); software used to prepare material for publication: *SHELXL97*.

## Supplementary Material

Crystal structure: contains datablock(s) I, global. DOI: 10.1107/S1600536811025414/tk2760sup1.cif

Structure factors: contains datablock(s) I. DOI: 10.1107/S1600536811025414/tk2760Isup2.hkl

Supplementary material file. DOI: 10.1107/S1600536811025414/tk2760Isup3.mol

Additional supplementary materials:  crystallographic information; 3D view; checkCIF report

## Figures and Tables

**Table 1:** Selected bond lengths (Å)

Cu1—Cl1	2.2306 (15)
Cu1—Cl2	2.5353 (16)
Cu1—N1	2.038 (3)
Cu1—N2	1.989 (2)
Cu1—N4	2.011 (2)

**Table 2:** Hydrogen-bond geometry (Å, °) *Cg*1 and *Cg*2 are the centroids of the N4,N5,C16–C19 and N1,C15,C28,C29–C31 rings, respectively.

*D*—H⋯*A*	*D*—H	H⋯*A*	*D*⋯*A*	*D*—H⋯*A*
O1—H1⋯Cl2^i^	0.84	2.36	3.144 (3)	155
O3—H3a⋯N8^ii^	0.84	1.96	2.773 (4)	164
C2—H2⋯Cl1^iii^	0.95	2.78	3.683 (4)	158
C32—H32⋯Cl2^iv^	0.95	2.64	3.480 (3)	148
C33—H33⋯Cl2^v^	0.95	2.80	3.694 (4)	158
C36—H36b⋯O3^ii^	0.98	2.26	3.225 (4)	169
C29—H29⋯O5	0.95	2.47	3.289 (5)	145
C20—H20b⋯*Cg*1^v^	0.98	2.58	3.381 (4)	139
C36—H36c⋯*Cg*2^iv^	0.98	2.81	3.646 (4)	144

Symmetry codes: (i) 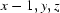; (ii) 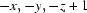; (iii) 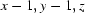; (iv) 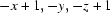; (v) 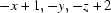.

**Fig. 1.**
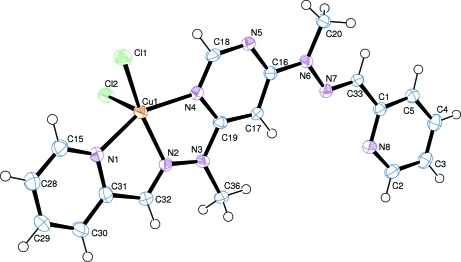
The molecular structure of the title compound drawn at 50% probability thermal ellipsoids. Solvent molecules are omitted for clarity.

**Fig. 2.**
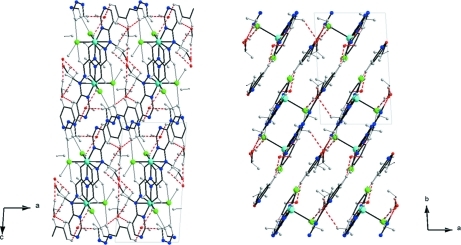
Packing of the CuII complex and constitutional solvent molecules; views in the direction of the crystallographic b- (left) and c-axes.

**Fig. 3.**
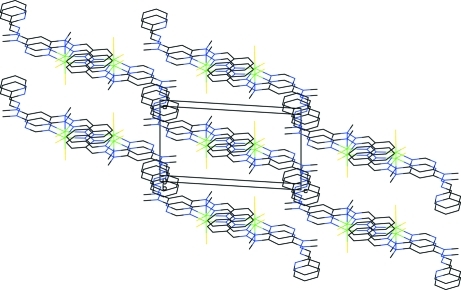
π–π Stacking between the CuII complexes; view in the direction of the c-axis.

## supplementary crystallographic information

### Comment

Heterocyclic *N*-containing ligands have been heavily exploited to create metallo-supramolecular structures such as helicates, grids, molecular ladders, *etc*. Lehn and co-workers recently reported (pyridin-2-ylmethylene)hydrazinyl)pyrimidine-based ligands and their Pb^II^, Zn^II^, Hg^II^ and La^III^ complexes (Stadler *et al.*, 2005; Stadler *et al.* 2006).

The asymmetric unit in the title compound contains a mononuclear Cu^II^ complex of 4,6-bis[(*E*)-1-methyl-2-(pyrindin-2-ylmethylene)hydrazinyl]pyrimidine (Fig. 1), two solvent methanol molecules and a water solvent molecule. The Cu^II^ atom is penta-coordinated by three N atoms of the organic ligand and two chloride atoms, Table 1. The coordination geometry of the central Cu^II^ is best described as distorted square pyramid. The structural distortion index (Addison *et al.*, 1984), τ, is 0.08 compared with an ideal τ value of 0.0 for a square pyramid. In this description, the N1 N2 N4 Cl2 atoms form the basal plane, and Cl1 occupies the apical position . The bond angles around the central Cu atom range between 78.04 (10)–159.90 (7)°. The configuration around both imine bonds is assigned to be *E*.

The crystal structure is stabilized by hydrogen bonds between the Cu^II^ complex and the constitutional solvent molecules, Table 2, and π–π interactions between the pyrimidine and pyridine rings of symmetry related molecules (Figs 2 & 3). The centroid-centroid distances of the pyrimidine···pyridine (symmetry code:-*x*, -*y*, 2 - *z*) and pyridine···pyridine (symmetry code: -*x*, -1 - *y*, 2 - *z*) interactions are 3.750 (3) and 3.850 (3) Å, respectively. In addition, weak C—H···π interactions are also observed, Table 2.

### Experimental

4,6-bis[*N*-Methyl-2-(pyrindin-2-ylmethylene)hydrazinyl]pyrimidine (0.007 g. 0.025 mmol) was dissolved in 5 ml of chloroform and 4.75 ml of ethanol. Then, 0.25 ml of an ethanolic 0.1 *M* copper(II) chloride dihydrate solution was added and the mixture was left for slow evaporation. Green blocks of the title compound were collected after 2 days. Yield: *ca* 75%.

### Refinement

All the hydrogen atoms were positioned geometrically (C—H = 0.95–0.98 Å; O—H = 0.84 Å) and were included in the refinement in the riding model approximation with *U*_iso_(H)= 1.2*U*_eq_(C) and 1.5*U*_eq_(O). The H atoms of O5 of the water molecule could not be located. The maximum and minimum residual electron density peaks of 1.20 and 0.64 eÅ^-3^, respectively, were located 0.07 Å and 0.67 Å from the O5 atom, respectively.

### Crystal data

**Table:**

[CuCl_2_(C_20_H_18_N_8_)]·2CH_4_O·H_2_O	*Z* = 2
*M**_r_* = 560.94	*F*(000) = 578
Triclinic, *P*1	*D*_x_ = 1.551 Mg m^−^^3^
Hall symbol: -P 1	Mo *K*α radiation, λ = 0.71075 Å
*a* = 7.430 (5) Å	Cell parameters from 4091 reflections
*b* = 11.627 (8) Å	θ = 1.5–31.2°
*c* = 14.026 (9) Å	µ = 1.17 mm^−^^1^
α = 95.848 (7)°	*T* = 116 K
β = 93.477 (13)°	Block, green
γ = 92.920 (9)°	0.30 × 0.25 × 0.10 mm
*V* = 1201.2 (14) Å^3^	

### Data collection

**Table:**

Rigaku Saturn724 diffractometer	7050 independent reflections
Radiation source: fine-focus sealed tube	4093 reflections with *I* > 2σ(*I*)
graphite	*R*_int_ = 0.091
Detector resolution: 28.5714 pixels mm^-1^	θ_max_ = 31.0°, θ_min_ = 2.8°
φ and ω scans	*h* = −10→10
Absorption correction: multi-scan (*CrystalClear*; Rigaku, 2008)	*k* = −16→16
*T*_min_ = 0.786, *T*_max_ = 1.000	*l* = −20→20
26094 measured reflections	

### Refinement

**Table:**

Refinement on *F*^2^	Primary atom site location: structure-invariant direct methods
Least-squares matrix: full	Secondary atom site location: difference Fourier map
*R*[*F*^2^ > 2σ(*F*^2^)] = 0.047	Hydrogen site location: inferred from neighbouring sites
*wR*(*F*^2^) = 0.104	H-atom parameters constrained
*S* = 0.82	*w* = 1/[σ^2^(*F*_o_^2^) + (0.0252*P*)^2^] where *P* = (*F*_o_^2^ + 2*F*_c_^2^)/3
7050 reflections	(Δ/σ)_max_ = 0.001
311 parameters	Δρ_max_ = 1.20 e Å^−^^3^
0 restraints	Δρ_min_ = −0.64 e Å^−^^3^

### Special details

**Table:**

Geometry. All s.u.'s (except the s.u. in the dihedral angle between two l.s. planes) are estimated using the full covariance matrix. The cell s.u.'s are taken into account individually in the estimation of s.u.'s in distances, angles and torsion angles; correlations between s.u.'s in cell parameters are only used when they are defined by crystal symmetry. An approximate (isotropic) treatment of cell s.u.'s is used for estimating s.u.'s involving l.s. planes.
Refinement. Refinement of *F*^2^ against ALL reflections. The weighted *R*-factor *wR* and goodness of fit *S* are based on *F*^2^, conventional *R*-factors *R* are based on *F*, with *F* set to zero for negative *F*^2^. The threshold expression of *F*^2^ > 2σ(*F*^2^) is used only for calculating *R*-factors(gt) *etc*. and is not relevant to the choice of reflections for refinement. *R*-factors based on *F*^2^ are statistically about twice as large as those based on *F*, and *R*- factors based on ALL data will be even larger.

### Fractional atomic coordinates and isotropic or equivalent isotropic displacement parameters (Å^2^)

**Table:**

	*x*	*y*	*z*	*U*_iso_*/*U*_eq_	
Cu1	0.51669 (5)	0.23008 (3)	0.67322 (2)	0.02337 (11)	
Cl1	0.60610 (11)	0.40752 (6)	0.74048 (5)	0.03137 (19)	
Cl2	0.80299 (10)	0.12043 (6)	0.67078 (5)	0.02719 (17)	
N8	−0.0738 (3)	−0.3088 (2)	0.95926 (17)	0.0257 (6)	
N7	0.1465 (3)	−0.1045 (2)	0.99451 (17)	0.0224 (5)	
N6	0.2460 (3)	−0.00054 (19)	1.01525 (16)	0.0212 (5)	
N5	0.4077 (3)	0.15083 (19)	0.95650 (16)	0.0218 (5)	
N4	0.4243 (3)	0.16279 (19)	0.78868 (16)	0.0212 (5)	
N1	0.5074 (3)	0.2629 (2)	0.53308 (16)	0.0235 (5)	
N2	0.3547 (3)	0.09759 (19)	0.61299 (16)	0.0210 (5)	
N3	0.2856 (3)	0.02344 (19)	0.67263 (17)	0.0236 (5)	
C1	−0.0123 (4)	−0.2672 (2)	1.0503 (2)	0.0222 (6)	
C2	−0.1852 (4)	−0.4043 (2)	0.9493 (2)	0.0298 (7)	
H2	−0.2286	−0.4348	0.8862	0.036*	
C3	−0.2412 (4)	−0.4615 (3)	1.0258 (2)	0.0306 (7)	
H3	−0.3217	−0.5284	1.0149	0.037*	
C4	−0.1776 (4)	−0.4193 (3)	1.1175 (2)	0.0308 (7)	
H4	−0.2136	−0.4565	1.1711	0.037*	
C5	−0.0597 (4)	−0.3212 (2)	1.1308 (2)	0.0264 (7)	
H5	−0.0122	−0.2915	1.1933	0.032*	
C15	0.5832 (4)	0.3533 (3)	0.4945 (2)	0.0284 (7)	
H15	0.6540	0.4107	0.5356	0.034*	
C16	0.3030 (4)	0.0512 (2)	0.9371 (2)	0.0205 (6)	
C17	0.2558 (4)	0.0018 (2)	0.84227 (19)	0.0205 (6)	
H17	0.1839	−0.0686	0.8288	0.025*	
C18	0.4602 (4)	0.2000 (2)	0.8816 (2)	0.0224 (6)	
H18	0.5322	0.2704	0.8951	0.027*	
C19	0.3201 (4)	0.0616 (2)	0.7704 (2)	0.0211 (6)	
C20	0.2746 (4)	0.0561 (2)	1.11368 (19)	0.0246 (6)	
H20A	0.3352	0.1328	1.1128	0.037*	
H20B	0.3500	0.0091	1.1522	0.037*	
H20C	0.1578	0.0644	1.1418	0.037*	
C28	0.5623 (4)	0.3661 (3)	0.3978 (2)	0.0312 (7)	
H28	0.6165	0.4318	0.3733	0.037*	
C29	0.4617 (4)	0.2825 (3)	0.3368 (2)	0.0321 (7)	
H29	0.4466	0.2899	0.2700	0.039*	
C30	0.3827 (4)	0.1872 (3)	0.3747 (2)	0.0299 (7)	
H30	0.3135	0.1282	0.3344	0.036*	
C31	0.4081 (4)	0.1808 (3)	0.4734 (2)	0.0252 (7)	
C32	0.3240 (4)	0.0875 (2)	0.5211 (2)	0.0237 (6)	
H32	0.2526	0.0247	0.4869	0.028*	
C33	0.1001 (3)	−0.1588 (2)	1.0661 (2)	0.0212 (6)	
H33	0.1396	−0.1281	1.1297	0.025*	
C36	0.1671 (4)	−0.0758 (2)	0.6330 (2)	0.0250 (6)	
H36A	0.0583	−0.0487	0.6013	0.037*	
H36B	0.1328	−0.1212	0.6849	0.037*	
H36C	0.2304	−0.1243	0.5861	0.037*	
O1	0.0092 (3)	0.26564 (19)	0.52994 (16)	0.0421 (6)	
H1	−0.0750	0.2342	0.5572	0.063*	
C37	0.0780 (4)	0.3696 (3)	0.5842 (3)	0.0458 (9)	
H37A	−0.0173	0.4030	0.6217	0.069*	
H37B	0.1195	0.4247	0.5407	0.069*	
H37C	0.1794	0.3531	0.6277	0.069*	
O3	−0.0217 (4)	0.24581 (19)	0.21711 (17)	0.0615 (8)	
H3A	0.0257	0.2578	0.1659	0.092*	
C38	−0.0030 (4)	0.3467 (3)	0.2806 (2)	0.0374 (8)	
H38A	−0.0155	0.3271	0.3462	0.056*	
H38B	−0.0968	0.3988	0.2638	0.056*	
H38C	0.1163	0.3850	0.2762	0.056*	
O5	0.6024 (5)	0.2970 (3)	0.1203 (2)	0.0990 (11)	

### Atomic displacement parameters (Å^2^)

**Table:**

	*U*^11^	*U*^22^	*U*^33^	*U*^12^	*U*^13^	*U*^23^
Cu1	0.0281 (2)	0.0221 (2)	0.01938 (19)	−0.00223 (15)	0.00150 (15)	0.00127 (14)
Cl1	0.0424 (5)	0.0232 (4)	0.0269 (4)	−0.0065 (3)	−0.0001 (3)	0.0000 (3)
Cl2	0.0285 (4)	0.0287 (4)	0.0238 (4)	0.0021 (3)	0.0010 (3)	−0.0002 (3)
N8	0.0256 (14)	0.0267 (14)	0.0242 (13)	0.0000 (11)	0.0023 (11)	−0.0005 (11)
N7	0.0199 (13)	0.0239 (13)	0.0233 (13)	0.0009 (10)	0.0029 (10)	0.0013 (10)
N6	0.0232 (13)	0.0205 (12)	0.0195 (12)	0.0000 (10)	0.0023 (10)	0.0004 (10)
N5	0.0210 (13)	0.0221 (13)	0.0217 (13)	−0.0005 (10)	0.0017 (10)	0.0003 (10)
N4	0.0203 (13)	0.0211 (12)	0.0221 (12)	−0.0015 (10)	0.0025 (10)	0.0022 (10)
N1	0.0230 (14)	0.0268 (13)	0.0212 (13)	−0.0004 (11)	0.0023 (10)	0.0049 (11)
N2	0.0217 (13)	0.0192 (12)	0.0212 (12)	−0.0018 (10)	0.0035 (10)	−0.0013 (10)
N3	0.0284 (15)	0.0221 (13)	0.0190 (12)	−0.0042 (11)	0.0017 (10)	−0.0012 (10)
C1	0.0205 (16)	0.0222 (15)	0.0239 (15)	0.0029 (12)	0.0034 (12)	0.0000 (12)
C2	0.0251 (18)	0.0288 (17)	0.0336 (18)	−0.0020 (14)	−0.0005 (14)	−0.0023 (14)
C3	0.0274 (18)	0.0231 (16)	0.041 (2)	−0.0029 (13)	0.0035 (15)	0.0037 (14)
C4	0.0292 (18)	0.0286 (17)	0.0364 (19)	−0.0004 (14)	0.0059 (14)	0.0110 (15)
C5	0.0236 (17)	0.0302 (17)	0.0252 (16)	0.0008 (13)	0.0005 (13)	0.0032 (13)
C15	0.0281 (18)	0.0277 (17)	0.0289 (17)	−0.0002 (13)	0.0033 (14)	0.0002 (14)
C16	0.0185 (15)	0.0218 (14)	0.0212 (14)	0.0030 (12)	0.0022 (12)	0.0008 (12)
C17	0.0172 (15)	0.0222 (14)	0.0215 (15)	−0.0027 (11)	0.0010 (11)	0.0009 (12)
C18	0.0205 (16)	0.0185 (14)	0.0264 (15)	−0.0025 (12)	−0.0007 (12)	−0.0031 (12)
C19	0.0201 (15)	0.0223 (15)	0.0204 (14)	0.0032 (12)	0.0004 (12)	−0.0016 (12)
C20	0.0304 (17)	0.0228 (15)	0.0199 (15)	−0.0025 (13)	0.0017 (12)	0.0006 (12)
C28	0.0331 (19)	0.0321 (18)	0.0295 (17)	0.0005 (14)	0.0053 (14)	0.0082 (14)
C29	0.038 (2)	0.0390 (19)	0.0200 (15)	−0.0006 (15)	0.0019 (14)	0.0062 (14)
C30	0.0351 (19)	0.0316 (17)	0.0219 (16)	−0.0001 (14)	0.0016 (14)	−0.0008 (13)
C31	0.0239 (16)	0.0298 (16)	0.0230 (15)	0.0041 (13)	0.0052 (12)	0.0047 (13)
C32	0.0229 (16)	0.0246 (15)	0.0227 (15)	−0.0020 (12)	−0.0004 (12)	0.0004 (12)
C33	0.0181 (15)	0.0238 (15)	0.0207 (14)	−0.0008 (12)	−0.0001 (12)	−0.0002 (12)
C36	0.0264 (17)	0.0249 (15)	0.0220 (15)	−0.0066 (13)	−0.0002 (12)	−0.0004 (12)
O1	0.0430 (15)	0.0446 (15)	0.0378 (14)	−0.0067 (12)	0.0104 (11)	−0.0002 (12)
C37	0.038 (2)	0.043 (2)	0.054 (2)	−0.0052 (17)	−0.0018 (18)	0.0018 (19)
O3	0.112 (2)	0.0336 (14)	0.0366 (15)	−0.0247 (15)	0.0352 (15)	−0.0096 (12)
C38	0.043 (2)	0.040 (2)	0.0297 (18)	0.0002 (16)	0.0058 (15)	0.0024 (15)
O5	0.115 (3)	0.101 (3)	0.079 (3)	−0.008 (2)	0.018 (2)	−0.001 (2)

### Geometric parameters (Å, °)

**Table:**

Cu1—Cl1	2.2306 (15)	C15—H15	0.9500
Cu1—Cl2	2.5353 (16)	C16—C17	1.410 (4)
Cu1—N1	2.038 (3)	C17—C19	1.377 (4)
Cu1—N2	1.989 (2)	C17—H17	0.9500
Cu1—N4	2.011 (2)	C18—H18	0.9500
N8—C2	1.342 (3)	C20—H20A	0.9800
N8—C1	1.362 (4)	C20—H20B	0.9800
N7—C33	1.294 (3)	C20—H20C	0.9800
N7—N6	1.380 (3)	C28—C29	1.384 (4)
N6—C16	1.381 (3)	C28—H28	0.9500
N6—C20	1.466 (3)	C29—C30	1.395 (4)
N5—C18	1.317 (3)	C29—H29	0.9500
N5—C16	1.356 (3)	C30—C31	1.397 (4)
N4—C18	1.337 (3)	C30—H30	0.9500
N4—C19	1.368 (3)	C31—C32	1.464 (4)
N1—C15	1.344 (3)	C32—H32	0.9500
N1—C31	1.358 (4)	C33—H33	0.9500
N2—C32	1.288 (3)	C36—H36A	0.9800
N2—N3	1.364 (3)	C36—H36B	0.9800
N3—C19	1.402 (3)	C36—H36C	0.9800
N3—C36	1.457 (3)	O1—C37	1.416 (4)
C1—C5	1.401 (4)	O1—H1	0.8400
C1—C33	1.465 (4)	C37—H37A	0.9800
C2—C3	1.392 (4)	C37—H37B	0.9800
C2—H2	0.9500	C37—H37C	0.9800
C3—C4	1.377 (4)	O3—C38	1.394 (4)
C3—H3	0.9500	O3—H3A	0.8399
C4—C5	1.391 (4)	C38—H38A	0.9800
C4—H4	0.9500	C38—H38B	0.9800
C5—H5	0.9500	C38—H38C	0.9800
C15—C28	1.381 (4)		
			
N2—Cu1—N4	78.01 (10)	C16—C17—H17	122.0
N2—Cu1—N1	79.32 (10)	N5—C18—N4	127.7 (3)
N4—Cu1—N1	155.14 (9)	N5—C18—H18	116.1
N2—Cu1—Cl1	159.91 (7)	N4—C18—H18	116.1
N4—Cu1—Cl1	99.43 (8)	N4—C19—C17	122.7 (3)
N1—Cu1—Cl1	98.34 (8)	N4—C19—N3	114.5 (2)
N2—Cu1—Cl2	95.54 (8)	C17—C19—N3	122.8 (3)
N4—Cu1—Cl2	95.41 (7)	N6—C20—H20A	109.5
N1—Cu1—Cl2	96.80 (7)	N6—C20—H20B	109.5
Cl1—Cu1—Cl2	104.54 (5)	H20A—C20—H20B	109.5
C2—N8—C1	116.9 (3)	N6—C20—H20C	109.5
C33—N7—N6	117.5 (2)	H20A—C20—H20C	109.5
C16—N6—N7	115.9 (2)	H20B—C20—H20C	109.5
C16—N6—C20	122.2 (2)	C15—C28—C29	119.4 (3)
N7—N6—C20	121.7 (2)	C15—C28—H28	120.3
C18—N5—C16	116.1 (2)	C29—C28—H28	120.3
C18—N4—C19	115.4 (2)	C28—C29—C30	119.2 (3)
C18—N4—Cu1	128.5 (2)	C28—C29—H29	120.4
C19—N4—Cu1	115.97 (18)	C30—C29—H29	120.4
C15—N1—C31	118.0 (3)	C29—C30—C31	118.1 (3)
C15—N1—Cu1	128.7 (2)	C29—C30—H30	120.9
C31—N1—Cu1	113.24 (18)	C31—C30—H30	120.9
C32—N2—N3	124.9 (2)	N1—C31—C30	122.6 (3)
C32—N2—Cu1	118.0 (2)	N1—C31—C32	114.9 (3)
N3—N2—Cu1	117.15 (17)	C30—C31—C32	122.5 (3)
N2—N3—C19	113.7 (2)	N2—C32—C31	114.5 (3)
N2—N3—C36	119.9 (2)	N2—C32—H32	122.8
C19—N3—C36	125.9 (2)	C31—C32—H32	122.8
N8—C1—C5	122.4 (3)	N7—C33—C1	120.9 (3)
N8—C1—C33	119.3 (3)	N7—C33—H33	119.6
C5—C1—C33	118.2 (3)	C1—C33—H33	119.6
N8—C2—C3	124.0 (3)	N3—C36—H36A	109.5
N8—C2—H2	118.0	N3—C36—H36B	109.5
C3—C2—H2	118.0	H36A—C36—H36B	109.5
C4—C3—C2	118.7 (3)	N3—C36—H36C	109.5
C4—C3—H3	120.7	H36A—C36—H36C	109.5
C2—C3—H3	120.7	H36B—C36—H36C	109.5
C3—C4—C5	119.1 (3)	C37—O1—H1	110.9
C3—C4—H4	120.5	O1—C37—H37A	109.5
C5—C4—H4	120.5	O1—C37—H37B	109.5
C4—C5—C1	118.9 (3)	H37A—C37—H37B	109.5
C4—C5—H5	120.5	O1—C37—H37C	109.5
C1—C5—H5	120.5	H37A—C37—H37C	109.5
N1—C15—C28	122.7 (3)	H37B—C37—H37C	109.5
N1—C15—H15	118.7	C38—O3—H3A	109.4
C28—C15—H15	118.7	O3—C38—H38A	109.5
N5—C16—N6	116.5 (2)	O3—C38—H38B	109.5
N5—C16—C17	122.1 (2)	H38A—C38—H38B	109.5
N6—C16—C17	121.4 (3)	O3—C38—H38C	109.5
C19—C17—C16	116.0 (3)	H38A—C38—H38C	109.5
C19—C17—H17	122.0	H38B—C38—H38C	109.5
			
C33—N7—N6—C16	175.9 (2)	Cu1—N1—C15—C28	−178.4 (2)
C33—N7—N6—C20	−9.5 (4)	C18—N5—C16—N6	−179.5 (2)
N2—Cu1—N4—C18	−178.6 (2)	C18—N5—C16—C17	1.2 (4)
N1—Cu1—N4—C18	−154.0 (2)	N7—N6—C16—N5	−177.2 (2)
Cl1—Cu1—N4—C18	−18.9 (2)	C20—N6—C16—N5	8.3 (4)
Cl2—Cu1—N4—C18	86.9 (2)	N7—N6—C16—C17	2.2 (4)
N2—Cu1—N4—C19	5.93 (18)	C20—N6—C16—C17	−172.4 (2)
N1—Cu1—N4—C19	30.6 (3)	N5—C16—C17—C19	−0.9 (4)
Cl1—Cu1—N4—C19	165.64 (18)	N6—C16—C17—C19	179.8 (2)
Cl2—Cu1—N4—C19	−88.61 (19)	C16—N5—C18—N4	−0.8 (4)
N2—Cu1—N1—C15	176.8 (3)	C19—N4—C18—N5	0.2 (4)
N4—Cu1—N1—C15	152.3 (2)	Cu1—N4—C18—N5	−175.3 (2)
Cl1—Cu1—N1—C15	17.1 (3)	C18—N4—C19—C17	0.1 (4)
Cl2—Cu1—N1—C15	−88.8 (2)	Cu1—N4—C19—C17	176.2 (2)
N2—Cu1—N1—C31	−2.32 (19)	C18—N4—C19—N3	−179.4 (2)
N4—Cu1—N1—C31	−26.8 (3)	Cu1—N4—C19—N3	−3.3 (3)
Cl1—Cu1—N1—C31	−162.08 (18)	C16—C17—C19—N4	0.3 (4)
Cl2—Cu1—N1—C31	92.1 (2)	C16—C17—C19—N3	179.7 (2)
N4—Cu1—N2—C32	172.7 (2)	N2—N3—C19—N4	−3.2 (3)
N1—Cu1—N2—C32	2.9 (2)	C36—N3—C19—N4	−174.6 (2)
Cl1—Cu1—N2—C32	88.0 (3)	N2—N3—C19—C17	177.3 (2)
Cl2—Cu1—N2—C32	−93.0 (2)	C36—N3—C19—C17	5.9 (4)
N4—Cu1—N2—N3	−7.87 (18)	N1—C15—C28—C29	−0.9 (5)
N1—Cu1—N2—N3	−177.6 (2)	C15—C28—C29—C30	0.3 (5)
Cl1—Cu1—N2—N3	−92.5 (3)	C28—C29—C30—C31	0.4 (5)
Cl2—Cu1—N2—N3	86.50 (18)	C15—N1—C31—C30	0.1 (4)
C32—N2—N3—C19	−172.1 (3)	Cu1—N1—C31—C30	179.3 (2)
Cu1—N2—N3—C19	8.5 (3)	C15—N1—C31—C32	−177.7 (2)
C32—N2—N3—C36	−0.1 (4)	Cu1—N1—C31—C32	1.6 (3)
Cu1—N2—N3—C36	−179.56 (18)	C29—C30—C31—N1	−0.6 (5)
C2—N8—C1—C5	0.9 (4)	C29—C30—C31—C32	177.0 (3)
C2—N8—C1—C33	−176.0 (2)	N3—N2—C32—C31	177.7 (2)
C1—N8—C2—C3	0.4 (4)	Cu1—N2—C32—C31	−2.9 (3)
N8—C2—C3—C4	−0.9 (5)	N1—C31—C32—N2	0.8 (4)
C2—C3—C4—C5	−0.1 (4)	C30—C31—C32—N2	−176.9 (3)
C3—C4—C5—C1	1.3 (4)	N6—N7—C33—C1	177.3 (2)
N8—C1—C5—C4	−1.7 (4)	N8—C1—C33—N7	−2.3 (4)
C33—C1—C5—C4	175.2 (3)	C5—C1—C33—N7	−179.3 (3)
C31—N1—C15—C28	0.7 (4)		

### Hydrogen-bond geometry (Å, °)

**Table:**

Cg1 and Cg2 are the centroids of the N4,N5,C16–C19 and N1,C15,C28,C29–C31 rings, respectively.

**Table:**

*D*—H···*A*	*D*—H	H···*A*	*D*···*A*	*D*—H···*A*
O1—H1···Cl2^i^	0.84	2.36	3.144 (3)	155
O3—H3a···N8^ii^	0.84	1.96	2.773 (4)	164
C2—H2···Cl1^iii^	0.95	2.78	3.683 (4)	158
C32—H32···Cl2^iv^	0.95	2.64	3.480 (3)	148
C33—H33···Cl2^v^	0.95	2.80	3.694 (4)	158
C36—H36b···O3^ii^	0.98	2.26	3.225 (4)	169
C29—H29···O5	0.95	2.47	3.289 (5)	145
C20—H20b···Cg1^v^	0.98	2.58	3.381 (4)	139
C36—H36c···Cg2^iv^	0.98	2.81	3.646 (4)	144

Symmetry codes: (i) *x*−1, *y*, *z*; (ii) −*x*, −*y*, −*z*+1; (iii) *x*−1, *y*−1, *z*; (iv) −*x*+1, −*y*, −*z*+1; (v) −*x*+1, −*y*, −*z*+2.
